# IMPACT OF VIBRATION ON TREMOR IN OLDER ADULTS WITH PARKINSON’S DISEASE

**DOI:** 10.1093/geroni/igac059.2228

**Published:** 2022-12-20

**Authors:** Ingrid Pretzer-Aboff, R K Elswick, Amanda Watson, Minglong Sun, Woosub Jung, Ken Koltermann, Gang Zhou, Leslie Cloud

**Affiliations:** Virginia Commonwealth University, Richmond, Virginia, United States; Virginia Commonwealth University, Richmond, Virginia, United States; University of Pennsylvania, Philadelphia, Pennsylvania, United States; College of William & Mary, Williamsburg, Virginia, United States; College of William & Mary, Williamsburg, Virginia, United States; College of William & Mary, Williamsburg, Virginia, United States; William & Mary, Williamsburg, Virginia, United States; Virginia Commonwealth University School of Medicine, Henrico, Virginia, United States

## Abstract

This was a randomized trial to explore the safety and efficacy of the RMBand (Resonate Forward LLC, Delaware) device to alleviate Parkinson’s Disease (PD) tremors. The RMBand applies vibration to the proximal arm and was worn on the side of tremor dominance. Thirty subjects with PD and associated tremors were randomized to receive high vs. low-frequency vibration. Tremor assessments occurred for 20 minutes before, during 20 minutes of continuous vibration, and for 20 minutes immediately post-vibration in a single 60-minute session. Assessments (MDS-UPDRS part 3 and FTM tremor scale) were performed during each of the 3 phases by blinded raters. A quantitative assessment of tremor was performed throughout the session using wearable sensors on the distal arms. Linear mixed models were used for group comparisons. No significant difference was observed between the high and low dose groups in the MDS-UPDRS part 3 or FTM tremor scale (p=0.83 and 0.48 respectively), which may be due to the crude nature of these scales. Quantitative wearable sensor data were used to assess total time with tremors during the pre-vibration, vibration, and post-vibration periods. Time with tremor during the pre-vibration period was significantly greater than that during and post-vibration (p< 0.0001) for both dose groups, suggesting that vibration therapy applied to the proximal arm may suppress PD tremor. No significant adverse events related to vibration therapy occurred. In conclusion, the RMBand appears safe and possibly effective for suppressing PD tremor. Further study is warranted.

